# Possible Role of Fibrinaloid Microclots in Postural Orthostatic Tachycardia Syndrome (POTS): Focus on Long COVID

**DOI:** 10.3390/jpm14020170

**Published:** 2024-01-31

**Authors:** Douglas B. Kell, Muhammed Asad Khan, Binita Kane, Gregory Y. H. Lip, Etheresia Pretorius

**Affiliations:** 1 Department of Biochemistry, Cell and Systems Biology, Institute of Systems, Molecular and Integrative Biology, Faculty of Health and Life Sciences, University of Liverpool, Crown St, Liverpool L69 7ZB, UK; binita.kane@mft.nhs.uk; 2 The Novo Nordisk Foundation Centre for Biosustainability, Building 220, Chemitorvet 200, Technical University of Denmark, 2800 Kongens Lyngby, Denmark; 3 Department of Physiological Sciences, Faculty of Science, Stellenbosch University, Stellenbosch Private Bag X1, Matieland 7602, South Africa; 4 Directorate of Respiratory Medicine, Manchester University Hospitals, Wythenshawe Hospital, Manchester M23 9LT, UK; asad.khan12@nhs.net; 5 Manchester University Foundation Trust and School of Biological Sciences, University of Manchester, Manchester M13 9PL, UK; 6 Liverpool Centre for Cardiovascular Science at University of Liverpool, Liverpool John Moores University and Liverpool Heart & Chest Hospital, Liverpool L14 3PE, UK; gregory.lip@liverpool.ac.uk; 7 Danish Center for Health Services Research, Department of Clinical Medicine, Aalborg University, 9220 Aalborg, Denmark

**Keywords:** fibrinaloid microclots, postural orthostatic tachycardia syndrome (POTS), Long COVID, TeamClots

## Abstract

Postural orthostatic tachycardia syndrome (POTS) is a common accompaniment of a variety of chronic, inflammatory diseases, including long COVID, as are small, insoluble, ‘fibrinaloid’ microclots. We here develop the argument, with accompanying evidence, that fibrinaloid microclots, through their ability to block the flow of blood through microcapillaries and thus cause tissue hypoxia, are not simply correlated with but in fact, by preceding it, may be a chief intermediary cause of POTS, in which tachycardia is simply the body’s exaggerated ‘physiological’ response to hypoxia. Similar reasoning accounts for the symptoms bundled under the term ‘fatigue’. Amyloids are known to be membrane disruptors, and when their targets are nerve membranes, this can explain neurotoxicity and hence the autonomic nervous system dysfunction that contributes to POTS. Taken together as a system view, we indicate that fibrinaloid microclots can serve to link POTS and fatigue in long COVID in a manner that is at once both mechanistic and explanatory. This has clear implications for the treatment of such diseases.

## 1. Introduction

### Orthostasis, Orthostatic Intolerance, and POTS

Human beings have evolved to maintain a largely erect posture [1] and can adopt it from recumbent poses. Orthostasis describes the (normal) physiological response used to counteract the potential fall in blood pressure when a person who has been lying down assumes the upright position. This tendency occurs because, in an adult, gravity causes a shift of some 300 to 800 mL of blood from the upper to the lower body. This orthostasis depends strongly on the autonomic nervous system.

However, if the system does not respond properly, there can be a significant decrease in the central blood pressure; common symptoms of such hypoperfusion are dizziness, lightheadedness, and syncope (fainting). The resulting intolerance of the upright posture is known as orthostatic intolerance (OI). When accompanied by a sustained postural drop in blood pressure (of more than 20 mmHg systolic or 10 mmHg diastolic [2]), the patient is diagnosed with orthostatic hypotension, which is a form of orthostatic intolerance (OI). Another variant of OI occurs when there is less of a fall in blood pressure, but the autonomic response leads instead to a rapid increase in heart rate (tachycardia). This is known as postural orthostatic tachycardia syndrome (POTS) (e.g., [3,4,5]). POTS is a manifestation of autonomic dysregulation and is clinically characterized as excessive tachycardia upon standing in the presence of symptomatic orthostatic intolerance. We recognize that POTS may be classified into subtypes such as neuropathic POTS and hyperadrenergic POTS; however, most of the papers we cite do not in fact make this distinction, and, for the present purposes, we avoid doing so as well, since our chief aim here was simply to suggest that there is, in general, significant evidence for the role of fibrinaloid microclots in POTS.

Although well known in other contexts for at least three decades [6,7] (see Table 1), with at least 500,000 cases in the USA alone [8,9,10], mostly in women (5:1) [5,9,11,12,13,14], POTS has emerged as a frequent symptom of both acute [15] and long COVID (e.g., [16,17,18,19,20,21] as part of the wider cardiovascular dysautonomia spectrum; see Table 1).

The management of POTS has been the subject of prior reviews and guidelines and is beyond the aims of the present study [90,91]. Our focus in this study was mainly on microclots as a plausible, mechanistic basis for POTS, especially in relation to long COVID.

## 2. The Normal Control of Heart Rate

**Figure 1:**
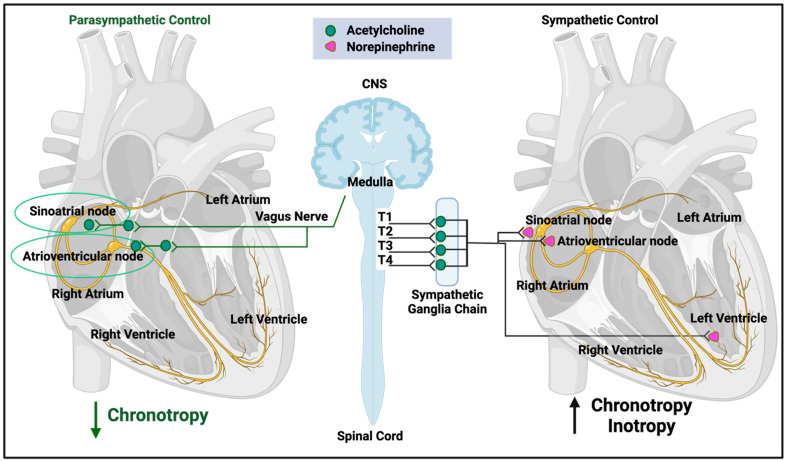
Autonomic nervous system regulation of heart function (after [92]). Created with BioRender.com. Access date: 26 November 2023.

Because of the general interest in POTS in long COVID and other affected communities, we include a very brief and high-level overview. The heart rate is controlled by many genetic and lifestyle factors (e.g., [92,93]), and the required kinds of understanding are both conceptual (e.g., the need to cater for the time-varying demands of tissue oxygenation) and mechanistic (e.g., the involvement of the endocrine and autonomic nervous systems). Our overview here is very far from being comprehensive, and our focus is necessarily on short-term control, where the autonomic nervous system is predominant (Figure 1, after [92]).

As summarized in Figure 1 (redrawn from [92]), both the sympathetic and parasympathetic branches of the autonomic nervous system are involved. The former is more involved in stress responses (often called ‘fight-or-flight’) and can release noradrenaline (norepinephrine) to increase heart rate, whilst the latter (often called ‘rest-and-digest’) underpins basal activity via the vagus nerve that can release acetylcholine to decrease heart rate relative to its base rate. Multiple control steps involve baroceptors that sense pressure and other receptors that respond to pH, hypoxia, and hypercapnia. In particular, under most conditions, the heart necessarily and appropriately responds to acute hypoxia by increasing heart rate (e.g., [94,95,96,97,98]).

## 3. Diagnosis of POTS

Most chronic, inflammatory diseases—as their name suggests—possess multiple common symptoms [99], while those such as long COVID characterized by subsets of multiple symptoms can easily be subclustered (e.g., [49,100,101,102]). The earlier definition of POTS comes from a very small study of 16 patients in 1993, of whom, interestingly, 7 were thought to have had previous viral infections [6,103]. Nowadays, for instance, the Canadian Cardiology Society has published a position paper describing a wider heterogenous range of clinical syndromes and a spectrum of orthostatic intolerance; they propose that discrete subtypes are identified over time, each with different underlying pathophysiological phenotypes that allow for specific targeted treatment [90]. However, for present purposes, in the case of POTS, both the high-level definition and the diagnosis are relatively straightforward, as they follow virtually from the name: heart rate is monitored for tachycardia (an increase in heart rate exceeding 30 beats per minute (bpm) within the initial 10 min of standing or head-up tilt (HUT)- or a ‘final’ value exceeding 120 bpm) as the individual changes their posture from horizontal to (more) vertical [5].

Differences can occur because the transition is commonly affected either by active standing or a passive ‘tilt table’ test [104,105,106,107]. The latter, which is somewhat more controlled and considered more reliable [108], commonly involves a ‘head-up tilt’ in which an individual is strapped to a horizonal table and commonly tilted to an angle of 60–80° [106,109], and heart rate and other measurements are performed. Transcranial doppler ultrasound may be used to detect blood flow [110]. It is recognized that such ‘provocative’ tests are of most value when individuals record similar symptoms to those that they normally experience [111]. For all events, the conceptual recognition of POTS is to be seen as reasonably straightforward [112,113]. It is important to recognize that the diagnostic criteria for heart rate changes are arbitrary and based on small case series, and that patients can have disabling OI and other symptoms of autonomic dysfunction without meeting the traditional cutoffs; this is no different in long COVID patients presenting with symptoms of POTS.

## 4. Occurrence and Comorbidities of POTS

Although we did not cover POTS (nor even autonomic dysfunction) in our earlier review of chronic, inflammatory diseases [99], the occurrence of POTS, which is highly heterogeneous [114], broadly mirrors the kinds of disease that we did mention there. Table 1 lists some of them, implying elements of a common origin. Of particular interest is the evidence for endothelial microvascular dysfunction [50], which can occur via the microclot-mediated blockage of red cell flow to tissues.

## 5. Dysautonomia

Autonomic dysfunction (dysautonomia) describes any malfunction in the autonomic nervous system, especially the vagus nerve [115,116], which is a key element in (but not synonymous with [117]) POTS, and the occurrence of dysautonomia broadly mirrors the diseases in which POTS is known to occur (Table 2).

## 6. Fatigue and POTS

Like POTS, fatigue is a common accompaniment of many acute and chronic inflammatory diseases. It is usually based on scoring questionnaires and thus lacks a crisp definition [135,136,137,138,139,140,141,142]. However, fatigue is generally used to cover a debilitating set of symptoms in which attempts to carry out what would normally be considered a very mild exertion are followed immediately by an inability to perform or to continue such exertions and a period in which extreme rest is required. In contrast to physiological ‘tiredness’, rest and sleep are not physically or mentally rejuvenating in fatigue. As noted in Table 1 [31,32,33,34,35,36,37], fatigue is a common accompaniment of POTS and—as we shall argue—likely has a main common cause.

## 7. The Role of Fibrinaloid Microclots in POTS

Although the origins of our discoveries that blood could clot into a very anomalous form lie earlier- in observations using the electron microscope (e.g., [143,144,145,146])- it was not until 2016 [147] that we determined using fluorescence microscopy that these anomalous forms were in fact amyloid in nature [148,149,150,151,152], that they could be induced by highly substoichiometric amounts of bacterial lipopolysaccharide [147], and that the electron and optical microscopies were congruent [153]. Essentially all the clots visible using fluorescence staining were those visible in the bright field [154,155]. The microclots were found to be particularly prevalent in diabetes [156,157,158] and in particular in both acute [158] and long COVID [159,160,161,162,163,164,165,166], where they could be induced by miniscule concentrations of the spike protein [167,168]. They were also much raised over those in controls in individuals with ME/CFS [169,170]. Note that the generation of fibrinaloid microclots is essentially instantaneous (on the timescale of normal clotting) (e.g., [147,167]), whereas the time taken to develop POTS is slower. This is at least consistent with a causative role of the earlier-appearing microclots in the generation of the later-appearing POTS.

**Figure 2:**
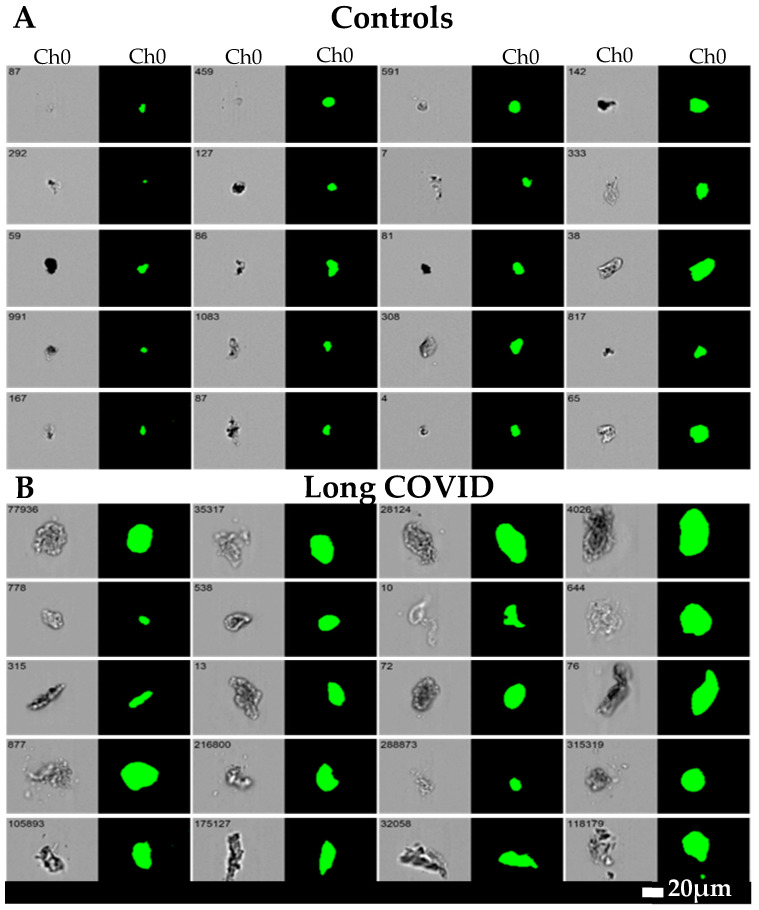
Microclot size distribution as seen with imaging flow cytometry (taken from [166]). Representative micrographs of microclots in (**A**) controls and (**B**) long COVID patients using an imaging flow cytometer. The brightfield images are displayed in Channel 1 (Ch01) and fluorescence intensity due to ThT binding in Channel 7 (Ch07). All images were captured using a 20x objective. The event number is displayed in the top-left corner of each image. NB: In these pictures, the POTS status of the individuals was not assessed.

Microclots differ from clots mostly by being considerably smaller (broadly in the range of 1–200 μm, mostly at the lower end) (see Figure 2) and by virtue both of the adoption of an amyloid form [148,159,161] and their entrapment of molecules such as α_2_-antiplasmin [163]. These and other properties [171] make them particularly resistant to fibrinolysis, so they are removed far less quickly than would normally be the case.

**Figure 3:**
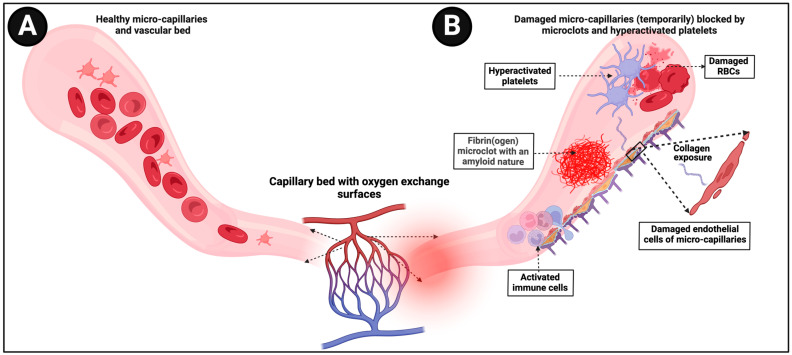
(**A**) Representation of healthy blood flow in microcapillaries (**B**) versus in an individual where damaged microcapillaries are (temporarily) blocked by microclots. Created with BioRender.com (accessed on 26 November 2023).

A straightforward consequence of these insoluble fibrinaloid microclots is that as blood flow pushes them along, they can block up microcapillaries, thereby inhibiting the flux of oxygen-carrying red blood cells and thus inducing tissue hypoxia. Sensing low tissue oxygen concentrations naturally (as when exercising) may induce tachycardia, and this would provide a very ready explanation of both POTS and the fatigue that is a common occurrence in both ME/CFS and long COVID (see Figure 3).

Other mechanisms for POTS in long COVID may include:

1. Relative hypovolemia secondary to inadequate peripheral vasoconstriction. This results in a reduction in stroke volume and cardiac output, causing the inhibition of tissue oxygen supply and the consequent compensatory tachycardia.
2. Small fiber neuropathy (SFN) has been well described in long COVID (e.g., [63,65,68,172]) and is a recognized cause of dysautonomia in the condition. SFN in long COVID can be driven by autoantibodies (already known to be associated with POTS and OH) or, potentially, by ischemia of the small fibres due to microclots.

## 8. The Role of Microclots in Fatigue

Just as the blocking of microcapillaries by microclots gives a ready explanation for POTS, it also gives a ready explanation for fatigue as tissues that rely on aerobic respiration for their normal function are deprived of oxygen. Specifically, the microclots vary widely in diameter, so they can migrate to those parts of the capillary bed where they can block the flow of red blood cells most effectively. Consequently, the affected tissues simply cannot perform their normal functions. While details vary for every individual, the existence and capillary-blocking behavior of the microclots also provide a simple and mechanistic explanation for the co-occurrence [31,32,33,35,36,37] of POTS and fatigue.

## 9. Relationship between Dysautonomia and Microclots

We know that molecules such as LPS (e.g., [147,149,150]) and the spike protein of SARS-CoV-2 (e.g., [154,158,159,163,164,165,166,167,173]) can cause microclots, such that any damage such molecules may cause to nerves may be indirect [174,175,176]. This said, it is reasonable that any damage to the membranes of nerves might be mediated via fibrinaloid microclots.

**Figure 4:**
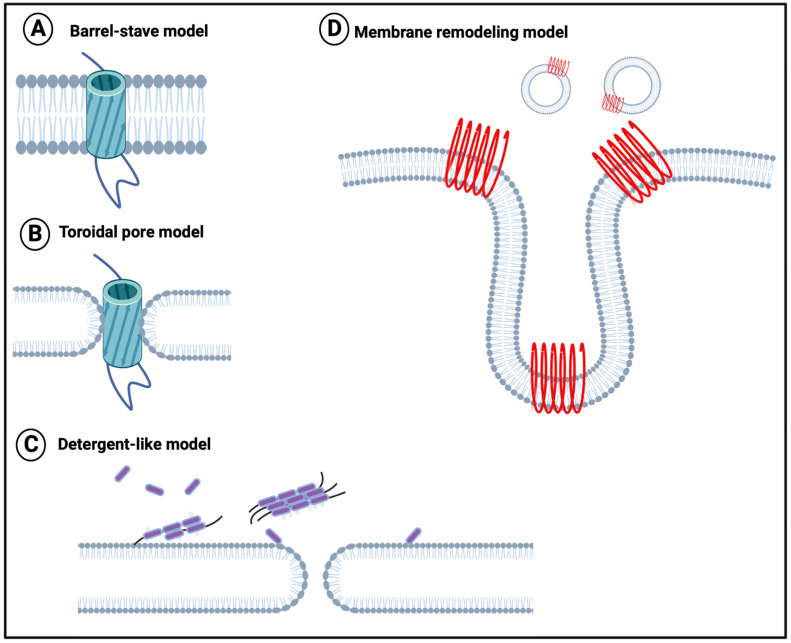
Membrane disruption models (redrawn from [201]). (**A**) The barrel-stave model suggests that proteins perpen-dicularly insert into the phospholipid bilayer plane, with the hydrophobic regions of protein oligomers contacting the hydrophobic interior of the membrane. (**B**) The toroidal pore model suggests that proteins insert perpendicular to the phospholipid bilayer, with the protein hydrophilic ends remaining in contact with the lipid head layer. (**C**) The deter-gent-like model, suggests that positively charged residues in the amyloidogenic protein bind to the membrane. (**D**) The membrane remodeling model suggests that membrane-bound peptides self-assemble into β-sheets that subsequently either form pores on the membrane surface (Pore formation model) or drag lipids out of the bilayer core (Detergent-like model). Created with BioRender.com (accessed on 26 November 2023).

To this end, although the direct experiments have not been performed with fibrinaloid microclots (nor is it easy to conduct them in vivo), it is at least worth repeating that it is well established that amyloid forms of proteins (including those binding cations [177]) generally can effect damage to all kinds of phospholipid membranes directly (e.g., [177,178,179,180,181,182,183,184,185,186,187,188,189,190,191,192,193,194,195,196,197,198,199,200,201,202]). A variety of mechanisms have been proposed, such as those in Figure 4 [201].

When the membrane in question is a nerve membrane, neurotoxicity (e.g., [198,203,204,205,206,207,208,209] (leading to autonomic nervous system dysfunction) may result.

## 10. Systems Overview and Conclusions

We established that fibrinaloid microclots accompany a variety of diseases in which POTS is frequently diagnosed, with fatigue as a frequent feature, as are autoantibodies [161], implying a similar kind of cause or at least intermediate. The microclots do seem to fulfill this intermediary role, as they also provide a realistic set of mechanisms. This said, it should be admitted that detailed temporal studies have not been conducted in animals (which may not even provide a decent model), while those studies that did test, e.g., SARS-CoV-2 infection, in human volunteers directly [210] did not seek to measure microclots.

Very recently, Wüst and colleagues showed a variety of defects in the skeletal muscle of long COVID patients, including both amyloid deposition and mitochondrial dysfunction [211]. Coupled with the evidence for lactate overproduction in both COVID-19 [212,213,214,215,216,217] and ME/CFS [133,218,219,220,221,222], both of which are associated with POTS (Table 1), this provides further evidence for a role of inadequate O_2_ uptake in these processes.

**Figure 5:**
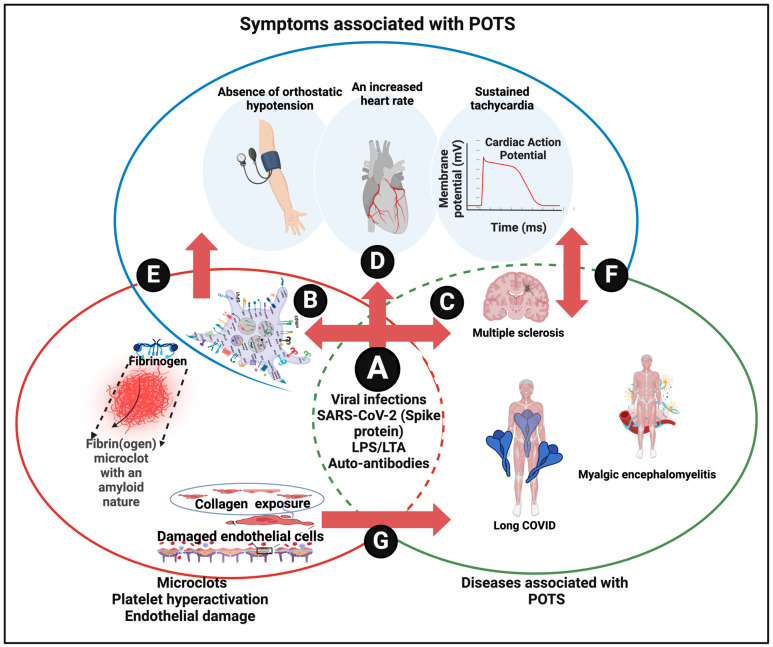
A system approach to defining dysautonomia. (A) Various causes of disease and symptoms resulting in vascular damage, microclots, and platelet hyperactivation (B) known to be involved in a variety of diseases (C) and in POTS (D). Similarly, vascular damage pathologies cause POTS (E) and other diseases (F), while POTS is found in various diseases (G). Created with BioRender.com (accessed on 26 November 2023).

The system biology diagram linking these high-level elements is given in Figure 5.

We conclude that the presence of fibrinaloid microclots can indeed significantly account for the symptoms of POTS associated with long COVID (and likely other syndromes), just as they can for other symptoms [159], post-exertional symptom exacerbation [160], and the generation of autoantibodies [161].

## Figures and Tables

**Table 1:** Some diseases and syndromes with which POTS is associated.

Disease, State, or Syndrome	Comments	Selected Reference(s)
Autoimmune disorders and Autoimmunity	Some strong associations	[16,22,23,24,25,26]
Cognitive function	Large amount of literature; improved by plasma exchange [27]	[27,28,29,30]
Fatigue		[31,32,33,34,35,36,37,38]
HPV or other antiviral vaccination	An example of induction by a viral protein	[39,40,41,42,43,44,45] but cf. [46]
Inflammation		[47]
Irritable bowel disease		[48]
Long COVID	A very common occurrence and a focus of our interest	[16,17,18,19,20,49,50,51,52,53,54,55,56,57,58,59,60,61,62,63,64,65,66,67,68]
Migraine		[69]
Multiple sclerosis	Now recognized as possibly caused by Epstein–Barr virus [70] (albeit much earlier evidence for an infectious origin existed [71,72], cf. [73,74]).	[75]
Myalgic encephalomyelitis/chronic fatigue syndrome (ME/CFS)	Is also usually a postviral disease and bears a number of similarities to long COVID [68,76,77,78,79]	[31,32,52,80,81,82,83,84]
Platelet delta granule storage pool deficiency	Causal direction unclear	[85]
Pregnancy	Many cardiovascular stresses accompany pregnancy, especially during hypertensive disorders [86,87]	[88,89]
Reviews		[22]

**Table 2:** Some diseases and syndromes in which dysautonomia is known to occur.

Disease, State, or Syndrome	Comments	Selected Reference(s)
Familial (monogenic)	Lesion in the IKBKAP gene	[118]
Long COVID		[57,60,62,63,67,76,119,120,121,122,123,124]
Multiple sclerosis		[125,126]
Myalgic encephalomyelitis/chronic fatigue syndrome		[76,82,119,127,128,129,130,131,132,133]
Parkinson’s disease		[134]
